# Functional connectivity correlates of infant and early childhood cognitive development

**DOI:** 10.1007/s00429-020-02027-4

**Published:** 2020-02-15

**Authors:** Muriel M. K. Bruchhage, Giang-Chau Ngo, Nora Schneider, Viren D’Sa, Sean C. L. Deoni

**Affiliations:** 1 Advanced Baby Imaging Lab, Women & Infants Hospital of RI, 555 Prospect St, Pawtucket, Providence, RI 20860 USA; 2 Department of Pediatrics, Warren Alpert Medical School at Brown University, 222 Richmond St, Providence, RI 02903 USA; 3 Nestlé Research, Vers-chez-les-Blanc, 1000 Lausanne, Switzerland; 4 Department of Radiology, Warren Alpert Medical School at Brown University, 222 Richmond St, Providence, RI 02912 USA; 5 Centre for Neuroimaging Science, Department for Psychiatry, Psychology and Neuroscience, King’s College London, London, UK

**Keywords:** Functional connectivity, MRI, Mullen, Motor, Language, Infant

## Abstract

Childhood is defined by the development of cognitive abilities as well as brain growth and function. While prior neuroimaging studies have investigated early development fragmentally, we studied the typical development of functional network connectivity continuously from infancy to childhood (average of 24 months) in 196 singleton term born children, as well as their emergence with age and visual, motor, and language abilities as assessed using the Mullen Scales of Early Learning. We demonstrate a cross-age shift to networks linked to higher-order cognitive processes, paralleling previous findings about developmental courses of functional connectivity networks. When investigating skill associations with functional connectivity independent of age, we revealed distinct network connectivity patterns for visual, motor, and language skills as each of them become more and more refined along childhood development. Specifically, the amount of functional networks recruited increases with skill complexity, with an exceeding involvement of higher order networks enabling daily maintenance and coordination of cognitive functions. Further, both motor and language network connectivity patterns overlapped in network connectivity patterns for the default mode, visual, salience, and dorsal attention networks, possibly implicating their overarching contribution to each other’s and higher cognitive development.

**Electronic supplementary material:**

The online version of this article (10.1007/s00429-020-02027-4) contains supplementary material, which is available to authorized users.

## Introduction

Infancy and early childhood are periods of rapid brain and cognitive development. This developmental period is defined by early motor and language milestones, which are facilitated by dynamic changes in underlying and sub-serving structural and functional brain networks (Silbereis et al. 2016). Structurally, processes such as myelination, dendritic growth, and synaptic pruning help to establish rapid and efficient communication pathways within and across neural networks. Through increased messaging speed and synchrony, these structural changes help drive the maturation of functional networks, which may incorporate several structurally connected brain regions that work together to perform cognitive tasks. Further, the topology and organization of functional networks may change with age and cognitive development, as specificity and specialization of related brain regions increases.

Functional networks, assessed using resting state functional connectivity imaging (rsFMRI), appear to be present already during prenatal brain development (Perani et al. 2011). Throughout infancy and childhood, these networks are highly dynamic and flexibly interact with each other and display differential developmental trajectories with age (Marrus et al. 2018), mirroring the temporal pattern of emerging cognitive function. Examining the topology of networks through both longitudinal studies and cross-sectional comparisons between infants and adults suggest a general evolution from local to distributed organization (Power et al. 2011). To lay the foundation for the development of more complex cognitive skills, essential functions need to be present very early during development. For example, one of the first functions to develop in utero is visual reception. To enable this, visual networks are present and functioning at birth, and amongst the first to reach adult-like status (Gao et al. 2017).

Paralleling cognitive skill development, motor and language networks develop subsequently together with skill ability. For example, Lin et al. (2008) have shown that the strength and extent of sensorimotor networks increase more rapidly than those of visual networks throughout early childhood (assessed at ages 2 weeks, 1 year, and 2 years) setting the foundation for the development of more complex skills. Investigating networks associated with walking, Marrus et al. (2018) recently reported increasing connectivity in motor networks and ability with age (at ages 12 and 24 months), possibly implying a continuous functional connectivity relationship with motor abilities originating in early development (Marrus et al. 2018). Similarly, language networks are already established at birth and progressively mature towards left hemispheric functional dominance, a characteristic of adult networks (Perani et al. 2011).

Therefore, past studies have provided insight into how functional brain systems organize with age, in step with evolving and maturing cognitive functions. However, due to the age ranges and developmental periods investigated, there remains a fragmented view of the relationships between functional networks, cognitive development, and performance, particularly across early childhood (birth to 5 years of age; for example, Zielinski et al. 2010). It is especially important to describe typical cognitive functioning in this age range, as they are essential for the understanding and detection of abnormal development.

To address this gap, we report on the patterns of correlation between resting state functional network connectivity and measures of language, motor, and visual functioning in a large cohort of neuro-typically developing children, 3 months to 6 years of age. Using rsFMRI data acquired from 196 healthy children without major risk factors for developmental abnormalities, we show an expected trend of increasing functional network connectivity with age, with little differences between boys and girls. Next, exploring the correlation between cognitive skills assessed using the Mullen Scales of Early Learning (Mullen 1995), we found expected overlaps in anatomical specificity and domain ability. Specifically, visual network connectivity increased with visual reception scores, sensorimotor network connectivity increased with both gross and fine motor scores, and language network connectivity increased with both receptive and expressive language scores. We also show more dynamic connectivity patterns with higher order networks such as the default mode, attention, and salience networks. As our findings investigate and describe the distinct underlying functional network connectivity patterns underlying different motor and cognitive abilities acquired throughout infancy and early childhood, it has the potential for a landmark to detect subclinical early aberrant brain—behavior patterns.

## Materials and methods

### Participant demographics

Data used in this study were drawn from the ongoing BAMBAM (Brown university Assessment of Myelination and Behavioral development Across Maturation) study of neurotypical brain and cognitive development, based at Brown University and located in Providence, RI, USA. For overview, BAMBAM was designed as an accelerated-longitudinal study of a large community cohort of healthy children. Approximately half of the study cohort was enrolled between 2 and 8 months of age; and the remainder between 2 and 4 years of age. Depending on child age, study visits occur every 6 months (under age 2) or 12 months, and include multi-modal MRI, performance, and parent reported measures of cognitive and behavioral functioning, anthropometry, and biospecimen collection. Participants were recruited with a wide range of different approaches, including online and newspaper advertisements, flyers, as well as referrals from pediatric hospitals.

Children with known major risk factors for developmental abnormalities at enrolment were excluded. Exclusion criteria included: in utero alcohol, cigarette or illicit substance exposure; preterm (< 37 weeks gestation) birth; small for gestational age or less than 1500 g; fetal ultrasound abnormalities; complicated pregnancy including preeclampsia, high blood pressure, or gestational diabetes; 5 min APGAR scores < 8; NCU admission; neurological disorder (e.g. head injury resulting in loss of consciousness, epilepsy); and psychiatric or learning disorder in the infant, parents or siblings (including maternal depression requiring medication in the year prior to pregnancy). In addition to screening at the time of enrollment, on-going screening for worrisome behaviors associated with neurodevelopmental disorders such as autism spectrum disorder using validated tools were performed to identify at-risk children and remove them from subsequent analysis. These included the modified checklist for autism (Robins et al. 1999), parent report measures (e.g. Child Behavior Checklist; Bilenberg et al. 1999), as well as medical and educational history data.

From the BAMBAM cohort, 196 out of 204 children were selected for analysis in this study (see details below), with each providing a single neuroimaging and neurocognitive dataset. With a 67% Caucasian and 33% non-white ratio, spanning families with income ranges lower than $10,000–$200,000 and above annual income with an mean average of $50,000–$69,999 real household income, our cohort is representative of the general US population (60.4% white and 39.6% non-white, average income of $61,372 as of 2017; Fontenot et al. 2018). Yearly household income was assessed in groups: (1) less than $10,000; (2) $10,000–$29,000; (3) $30,000–$49,000; (4) $50,000–$69,000; (5) $70,000–$89,000; (6) $90,000–$109,000; (7) $110,000–$149,000; (8) $150,000–$199,000; (9) $200,000 and more, as well as the option to prefer not to give information on family income.

Specific inclusion criteria were: (1) complete and high quality anatomical and rsFMRI dataset; (2) MRI data were acquired during natural sleep (so as to not mix awake and asleep rsFMRI data); (3) had a complete medical and family history that included birth outcome information; and (4) was a singleton full-term birth of healthy weight (more than 1500 g at birth). Participant demographics are provided in Table 1.

**Table 1:** Participant demographics

	Boys	Girls
Number	109	87
Age at scan in months	24.9 (18.1)	23.7 (18.3)
Gestation time in weeks	39.6 (1.0)	39.2 (1.3)
Birth weight in lbs	7.4 (1.0)	7.4 (1.0)
Maternal education	2.83 (3.0)	2.43 (2.9)
*Mullen*		
Early learning composite	96.83 (17.98)	105.94 (14.81)
Gross motor	18.01 (7.85)	17.45 (9.42)
Fine motor	22.65 (12.21)	22.49 (13.33)
Visual reception	25.07 (14.10)	24.95 (14.81)
Receptive language	21.97 (13.17)	22.21 (13.54)
Expressive language	21.02 (13.17)	20.99 (14.15)

Mean participant demographics with standard deviations in brackets. Average gestation time is given in weeks, birth weight is calculated in pounds, and maternal education was assessed with the Hollingshead scale

### MRI acquisition and functional connectivity processing

All neuroimaging data were acquired on a 3 T Siemens Trio scanner with a 12-channel head RF array. rsFMRI data were acquired during natural sleep with the following parameters: TE = 34 ms, TR = 2.5 s, flip angle = 80°, field of view = 24 × 24cm^2^, imaging matrix = 80 × 80, and 32 interleaved 3.6 mm slices (for a voxel resolution: 3 × 3 × 3.6mm^3^), BW = 751 Hz/pixel, and GRAPPA acceleration factor of 2. We acquired 132 volumes acquired for a total acquisition time of approximately 7:00 min. To achieve successful scanning without the use of sedatives, scans were scheduled around the child’s natural nap time. Once asleep, the child was transferred from one of the napping beds that included a bottom layer of custom made plexiglass that in turn enabled the child to be placed asleep onto the scanner bed. To remain in the sleep state, we used custom made patting going inside the bore to quiet the scanning noise, custom head phone pieces playing soothing rain sounds, pulse socks to monitor pulse and asleep behavior, as well as padding for the child to remain in a fixed position. At least one research assistant was in the scanner with the child watching at all times in addition to a camera facing the scanner.

T_1_-weighted anatomical data were also acquired using a magnetization prepared rapid acquisition gradient echo scan was acquired of each child with an isotropic voxel volume of 1.4 × 1.4 × 1.4 mm^3^. Sequence specific parameters were: TE = 6.9 ms; TR = 16 ms; inversion preparation time = 950 ms; flip angle = 15°; BW = 450 Hz/Pixel. The acquisition matrix and field of view were varied according to child head size to maintain a constant voxel volume and spatial resolution across all ages.

To extract connectivity values, the rsFMRI data were first preprocessed (including realignment, centering, motion correction, and scrubbing) with the CONN-fMRI toolbox for SPM 8 (Whitfield-Gabrieli et al. 2012) on MATLAB and registered to our child study template using FSL FLIRT (Smith et al. 2004) and ANTS (Avants et al. 2014). ROI-to-ROI connectivity analyses were performed, computing the correlation of spontaneous BOLD activity between network regions. This enabled us to determine differences in brain network connectivity with age as well as the individual Mullen raw domain scores (see below). A set of 32 anatomical ROIs were used in the network analysis (Whitfield-Gabrieli et al. 2012) and a reference of the distribution of the networks in the infant brain can be found in Figure S1. Using the implemented CompCor strategy (Behzadi et al. 2007), the effect of nuisance covariates including BOLD signal fluctuations from CSF, white matter and their derivatives, as well as the realignment parameter noises were reduced. Data were band-pass filtered (0.008 < *f* < 0.09 HZ). In addition, preprocessed images were visually inspected for remaining motion after data preprocessing, reducing the inclusion number from 204 to 196 children.

### Statistical analyses

We investigated the correlation of functional connectivity networks with age as well as the different Mullen raw scores using the CONN toolbox. Statistical analyses were performed for the following conditions: (1) correlation with child age (corrected to a 40-week gestation) for all participants; and (2) correlation with individual raw Mullen scores for visual reception, fine motor, gross motor, receptive language, and executive language, all corrected for child age. For analysis #2, all children were included in the analysis for fine motor, visual reception, and expressive and receptive language. Only children up to 2.5 years of age were included in the analysis for gross motor, since most children reach maximal score for this domain by 30 months (Table 1). All analysis for #1 and #2 were repeated for all children as well as for both sexes individually. Independent sample t-tests were used to determine differences between biological sex for Mullen raw scores using IBM SPSS version 24. For all analyses, significance was defined as *p* ≤ 0.05 false discovery rate (FDR) seed-level corrected for multiple comparisons.

### Mullen scales of early learning

Child cognitive development was assessed using the Mullen Scales of Early Learning (MSEL), a standardized and population normalized tool for assessing fine and gross motor, expressive and receptive language, and visual reception functioning in children from birth through 68 months of age. In addition to 5 raw scores and age-normalized domain-specific *T* scores (mean of 50, standard deviation of 7.5), the MSEL also provides a composite score that combines overall visual, motor, and language functioning.

## Results

### Functional network connectivity changes with child age

**Fig. 1:**
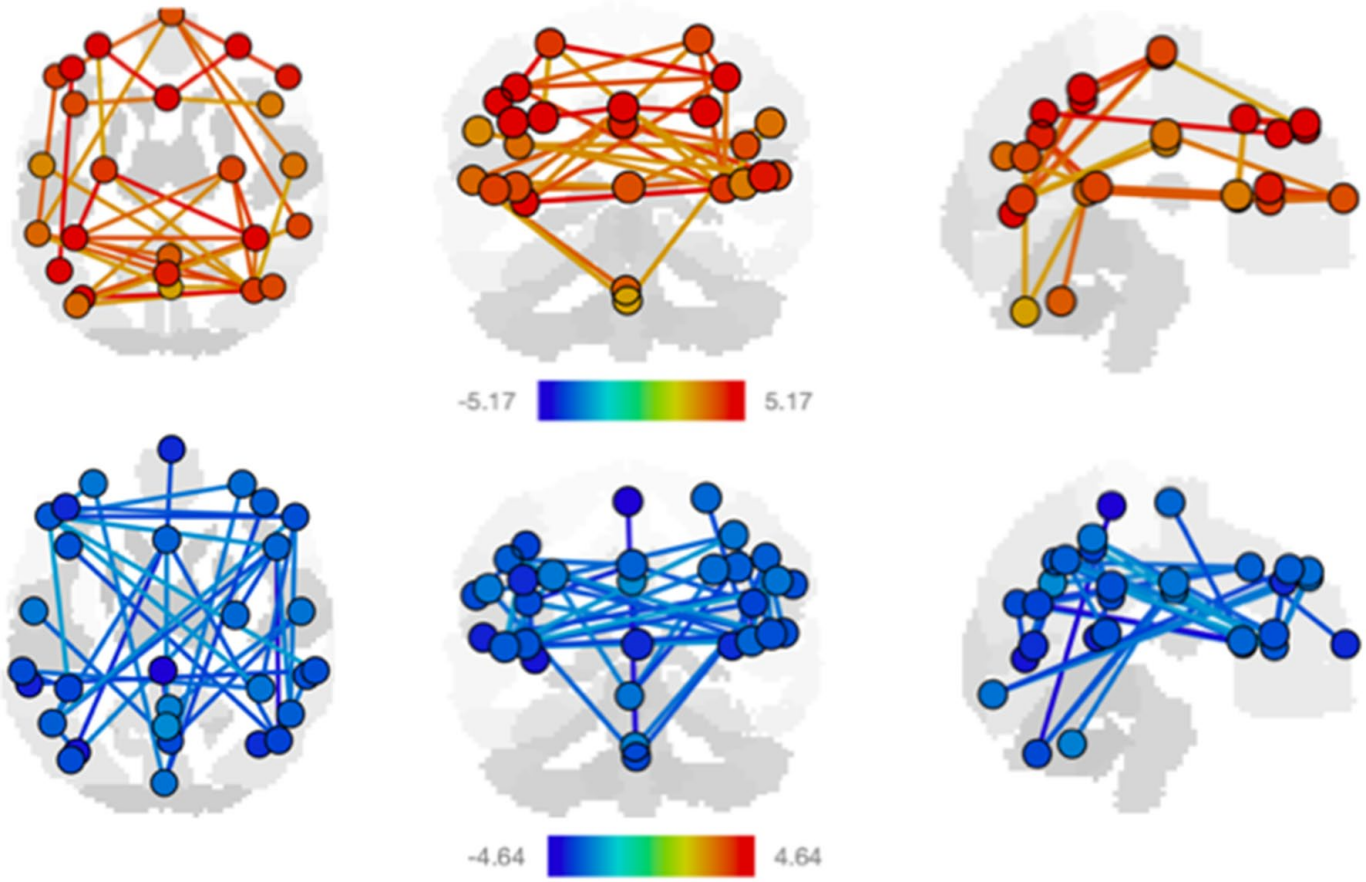
Significant resting state fMRI network connectivity changes with age divided into connectivity increases (top) and decreases (bottom) for all participants. Participant demographics can be found in Table 1. All analyses were corrected for biological sex, and *p* values were FDR corrected for multiple comparisons. *T* value ranges of region of interest effects are shown in a colour coded bar with yellow to red range indicating higher positive *t* values and green to blue range indicating higher negative *t* values

When boys and girls were combined, we found dynamic changes in connectivity across all functional networks when examining overall changes with age (Fig. 1, Table 2). Specifically, most changes with age were observed in language, salience, default mode, and attention networks, with a combination of down- and up-regulation across networks (Table 2). Exploring these age-related trends further and investigating potential differences in boys and girls, we found significant overlap (Table S2). Though there were subtle but distinct differences, specifically less connectivity in sensorimotor and language networks in girls when compared to boys, these results suggest unsurprisingly that the general networks underlying individual cognitive skills and behaviors do not differ in boys and girls, and that they develop in a consistent manner.

**Table 2:** Resting state fMRI network connectivity with age

Seed network	Seed region	Connected network	Connected region	*T*	*p* corr
Default mode	PCC	Default mode	LP (r.)	3.66	.0046
	PCC	Language	pST (l)	3.57	.0046
			pST (r.)	4.07	.0021
	LP (r.)	Default mode	LP (l.)	2.74	.0483
			PCC	3.66	.0046
		Salience	Anterior insula (l.)	− 3.40	.0084
			Anterior insula (r.)	− 4.64	.0002*
			ACC	− 3.26	.0013
		Cerebellar	Posterior	2.68	.0417
	MPFC	Sensorimotor	Lateral (r.)	3.25	.0085
		Language	pSTG (r.)	3.61	.0051
			pSTG (l.)	3.57	.0046
			IFG (r.)	4.09	.0020
			IFG (l.)	3.54	.0051
Sensorimotor	Superior	Cerebellar	Posterior	− 4.38	.0006*
	Lateral (r.)	Default mode	MPFC	3.25	.0212
		Cerebellar	Anterior	− 2.91	.0424
			Posterior	− 3.31	.0212
Visual	Lateral (l.)	Visual	lateral (r.)	5.17	.0000*
		Dorsal attention	IPS (r.)	3.73	.0039
			FEF (r.)	2.90	.0262
		Default mode	LP (l.)	− 3.50	.0060
		Frontoparietal	PPC (l.)	− 3.10	.0175
	Lateral (r.)	Visual	Lateral (l.)	5.17	.0000*
		Dorsal attention	IPS (l.)	3.99	.0014
			IPS (r.)	3.30	.0071
			FEF (l.)	2.68	.0357
			FEF (r.)	3.68	.0031
		Frontoparietal	PPC (r.)	− 3.49	.0046
		Sensorimotor	Lateral (l.)	2.89	.0225
	Occipital	Frontoparietal	LPFC (r.)	− 3.52	.0165
salience	SMG (l.)	Salience	IPS (l.)	− 3.37	.0280
			IPS (r.)	− 2.98	.0497
	SMG (r.)	Language	pSTG (l.)	− 3.46	.0203
	RPFC (l.)	Salience	ACC	4.48	.0004*
	RPFC (r.)	Salience	ACC	4.38	.0006*
	Anterior insula (l.)	Salience	ACC	3.32	.0164
		Default mode	LP (r.)	− 3.40	.0164
	Anterior insula (r.)	Salience	ACC	2.68	.0417
		Default mode	LP (r.)	− 4.64	.0002*
			LP (l.)	− 2.69	.0417
		Dorsal attention	FEF (r.)	− 3.39	.0108
		Frontoparietal	IPS (l.)	− 3.33	.0108
			IPS (r.)	− 2.71	.0417
	ACC	Salience	RPFC (l.)	4.48	.0003*
			RPFC (r.)	4.38	.0003*
			Anterior insula (l.)	3.32	.0066
			Anterior insula (r.)	2.68	.0284
		Default mode	LP (l.)	− 3.79	.0021
			LP (r.)	− 3.26	.0068
			MPFC	− 3.42	.0059
			PCC	− 2.67	.0284
		Frontoparietal	PPC (l.)	− 3.00	.0137
Dorsal attention	FEF (l.)	Dorsal attention	IPS (l.)	4.57	.0001*
			IPS (r.)	4.55	.0001*
	FEF (r.)	Dorsal attention	IPS (r.)	4.03	.0025
			IPS (l.)	3.45	.0065
		Visual	Lateral (r.)	3.68	.0046
			Lateral (l.)	2.90	.0262
		Salience	Anterior insula (r.)	− 3.39	.0065
	IPS (l.)	Dorsal attention	FEF (l.)	4.57	.0003*
			FEF (r.)	3.45	.0054
			IPS (r.)	3.83	.0018
		Visual	Lateral (r.)	3.99	.0014
		Salience	SMG (l.)	− 3.37	.0056
		Frontoparietal	PPC (l.)	− 2.82	.0273
	IPS (r.)	Dorsal attention	FEF (l.)	4.55	.0003*
			FEF (r.)	4.03	.0012
			IPS (l.)	3.83	.0018
		Visual	Lateral (l.)	3.73	.0010
			Lateral (r.)	3.30	.0071
		Language	IFG (r.)	− 3.20	.0083
		Salience	SMG (l.)	− 2.98	.0142
		Default mode	PCC	− 2.56	.0437
Frontoparietal	LPFC (l.)	Frontoparietal	PPC (l.)	4.68	.0002*
		Language	IFG (r.)	− 3.23	.0227
			IFG (l.)	− 3.07	.0257
	PPC (l.)	Frontoparietal	LPFC (l.)	4.68	.0002*
		Salience	Anterior insula	− 3.33	.0162
			ACC	− 3.00	.0240
		Visual	Lateral (l.)	− 3.10	.0233
		Dorsal attention	IPS (l.)	− 2.82	.0328
		Cerebellar	Anterior	2.75	.0338
	LPFC (r.)	Visual	Occipital	− 3.52	.0165
	PPC (r.)	Visual	lateral (r.)	− 3.49	.0185
Language	pSTG (r.)	Default mode	PCC	4.07	.0021
			MPFC	3.61	.0060
			LP (l.)	2.78	.0463
		Frontoparietal	PPC (r.)	− 2.92	.0405
	pSTG (l.)	Language	IFG (l.)	3.59	.0051
		Default mode	PCC	3.57	.0051
			MPFC	2.79	.0357
		Cerebellar	Anterior	3.49	.0051
		Salience	SMG (r.)	− 3.46	.0051
	IFG (r.)	Language	IFG (r.)	− 3.48	.0096
		Default mode	MPFC	4.09	.0020
		Frontoparietal	LPFC (l.)	− 3.23	.0124
			PPC (r.)	− 2.81	.0341
		Dorsal attention	IPS (r.)	− 3.20	.0124
	IFG (l.)	Language	pSTG (l.)	3.59	.0064
			IFG (r.)	− 3.48	.0064
		Default mode	MPFC	3.54	.0064
		Frontoparietal	LPFC (l.)	− 3.07	.0192
		Salience	RPFC (r.)	− 2.79	.0357
			RPFC (l.)	− 2.65	.0448
Cerebellum	Anterior	Language	pSTG (l.)	3.49	.0188
	Posterior	Sensorimotor	Superior	− 4.38	.0006*
			Lateral (r.)	− 3.31	.0006*
			Lateral (l.)	− 3.09	.0174

Significant resting state fMRI network connectivity age interactions with *T* and *p* values on the right. Seed networks and sub regions are displayed in the left two columns, and connected network connectivity regions in the middle. All analyses were corrected for biological sex, and *p* values were FDR corrected for multiple comparisons. *p* < 0.001 are highlighted with *

*MPFC* medial prefrontal cortex, *LP* lateral parietal cortex, *PCC* posterior cingulate cortex, *ACC* anterior cingular cortex, *RPFC* rostral prefrontal cortex, *SMG* supramarginal gyrus, *FEF* frontal eye field, *IPS* intraparietal sulcus, *LPFC* lateral prefrontal cortex, *PPC* posterior parietal cortex, *IFG* inferior frontal gyrus, *pSTG* posterior superior temporal gyrus

### Functional network connectivity correlates with Mullen development domains

**Fig. 2:**
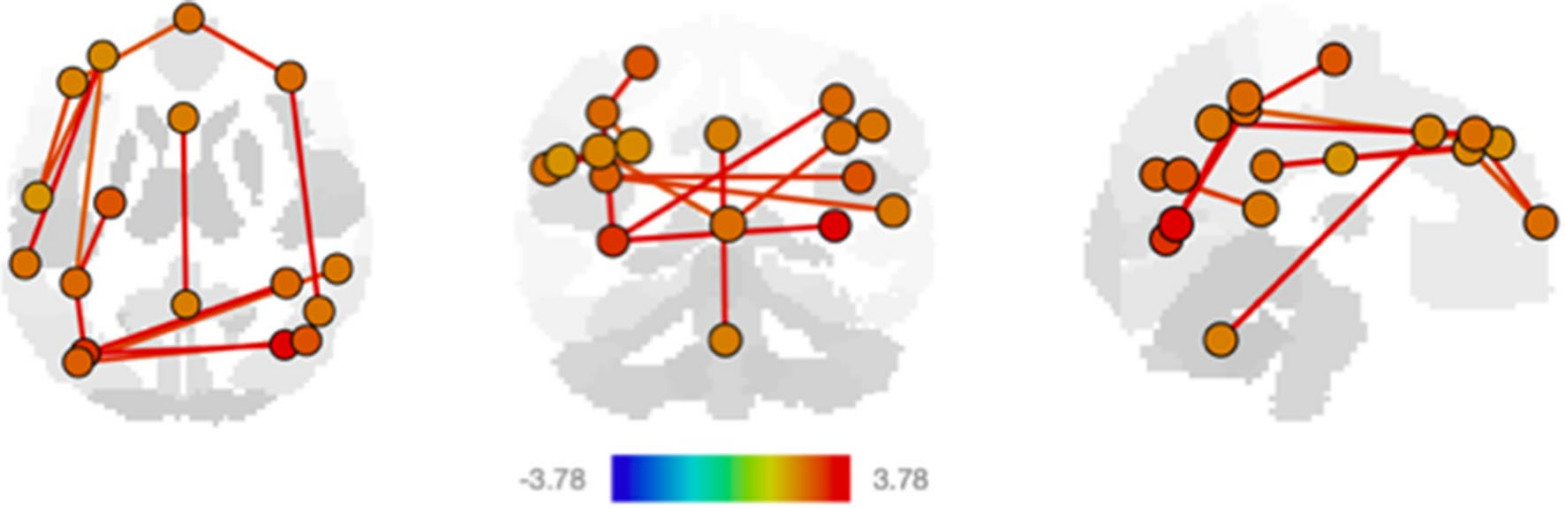
Significant resting state fMRI network connectivity changes with visual reception. All analyses were corrected for gestational age and biological sex, and *p* values were FDR corrected for multiple comparisons. *T* value ranges of region of interest effects are shown in a colour coded bar with yellow to red range indicating higher positive *t* values and green to blue range indicating higher negative *t* values

**Fig. 3:**
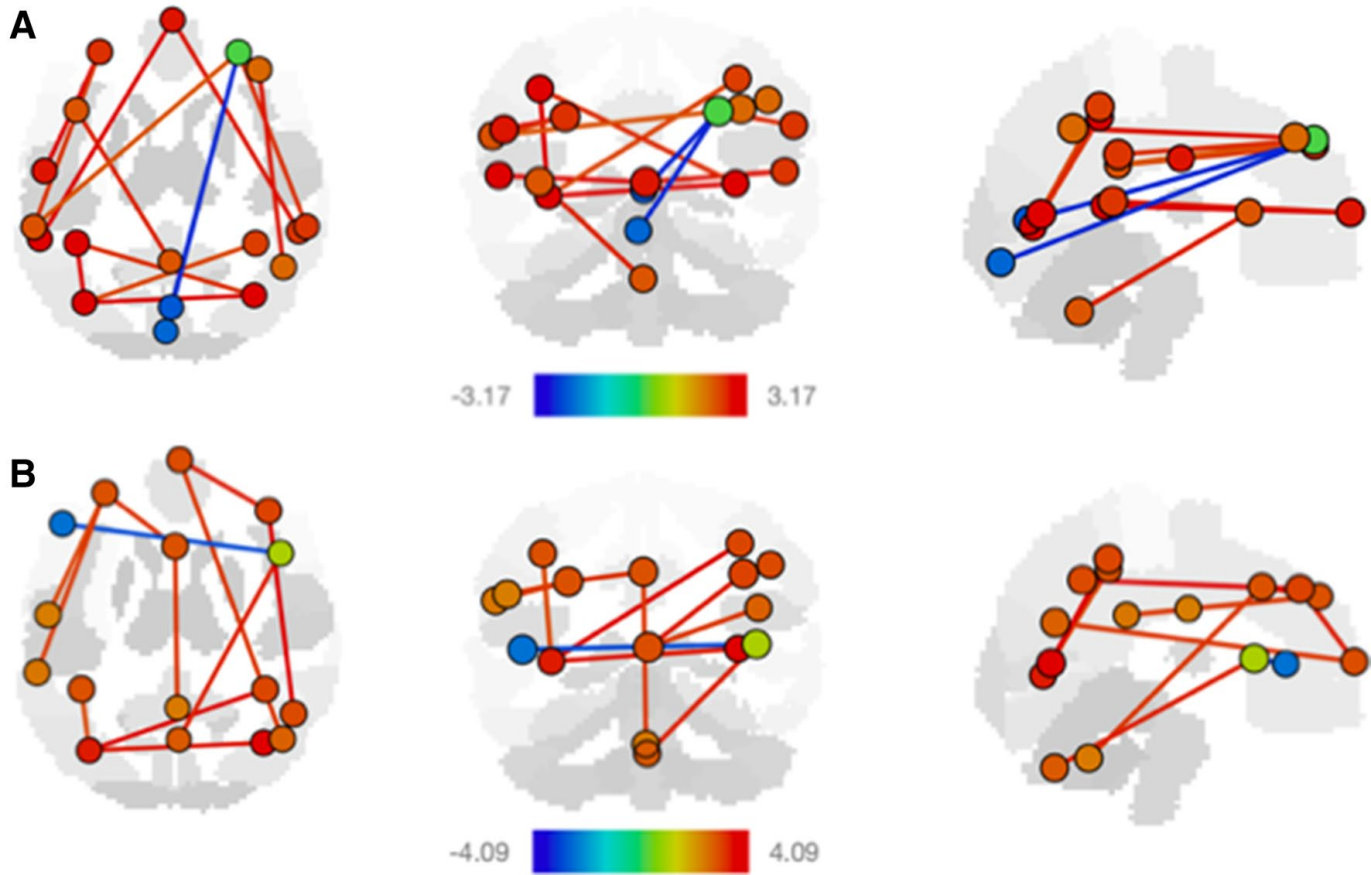
Significant resting state fMRI network connectivity changes with **a** gross motor and **b** fine motor ability. Participant demographics can be found in Table 1. All analyses were corrected for gestational age and biological sex, and *p* values were FDR corrected for multiple comparisons. *T* value ranges of region of interest effects are shown in a colour coded bar with yellow to red range indicating higher positive *t* values and green to blue range indicating higher negative *t* values

**Fig. 4:**
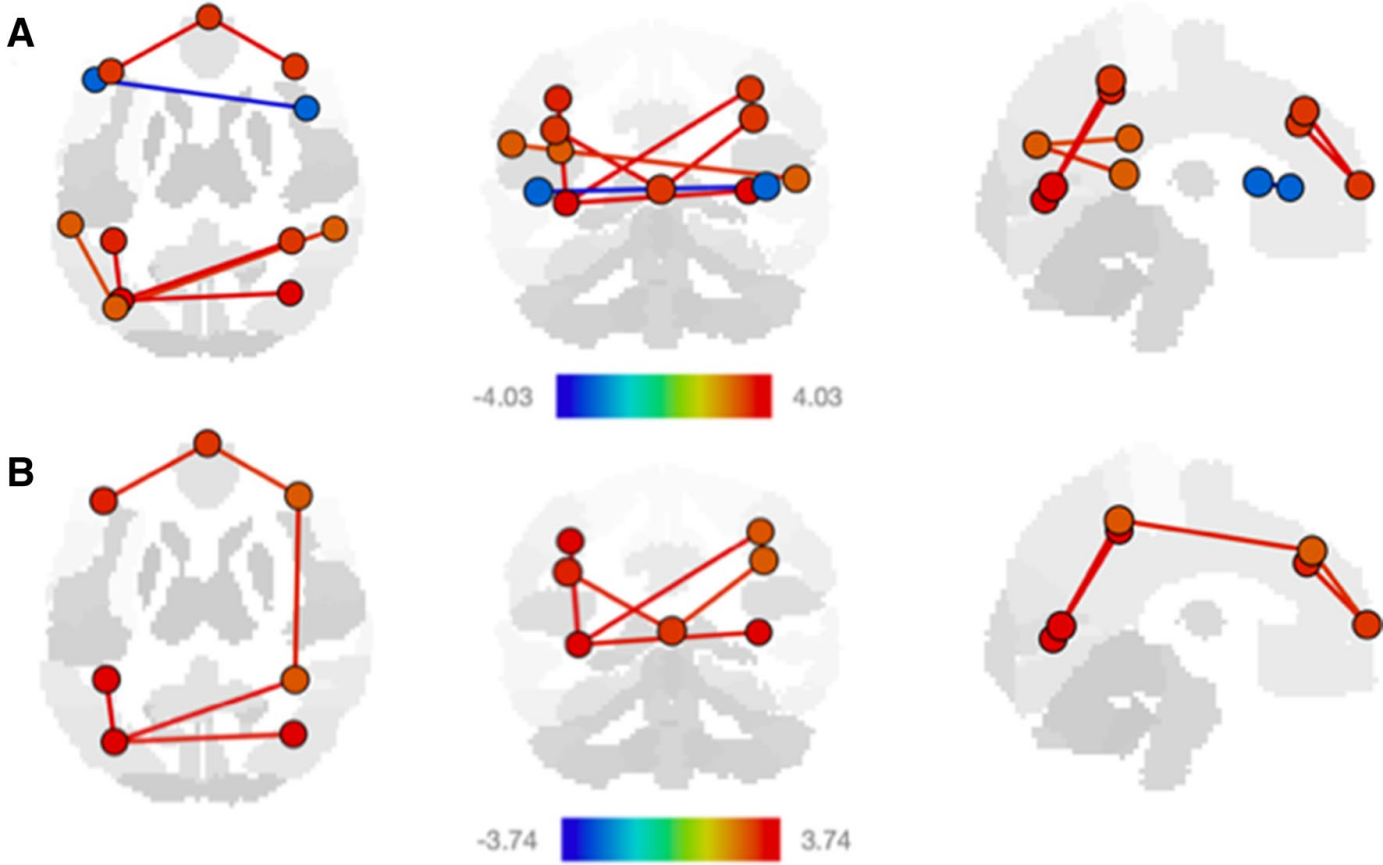
Significant resting state fMRI network connectivity changes with **a** receptive language and **b** executive language. Participant demographics can be found in Table 1. All analyses were corrected for gestational age and biological sex, and *p* values were FDR corrected for multiple comparisons. *T* value ranges of region of interest effects are shown in a colour coded bar with yellow to red range indicating higher positive *t* values and green to blue range indicating higher negative *t* values

Investigating connectivity changes associated with specific cognitive domains in our full child cohort, we found that improved visual, motor, and language functioning was associated with changes in connectivity across distinct functional networks (Figs. 2, 3, 4, Table 3). Specifically, visual reception was associated with increased connectivity in visual and dorsal attention networks, salience, frontoparietal, and cerebellar networks (Fig. 2, Table 3). Both increasing gross and fine motor ability independently were associated with increased default mode, visual, salience, dorsal attention, frontoparietal, and cerebellar network connectivity, while gross motor was additionally associated with sensorimotor and language network connectivity (Fig. 3, Table 3).

**Table 3:** Resting state fMRI network connectivity with Mullen scores

Seed network	Seed region	Connected network	Connected region	*T*	*p* corr
*(a) Gross motor*					
Default mode	MPFC	Language	pST (l)	3.58	.0146
			pST (r.)	3.31	.0185
Sensorimotor		Salience	RPFC (l.)	3.90	.0047
Visual	lateral (l.)	Visual	Lateral (r.)	3.77	.0074
		Dorsal attention	IPS (l.)	3.44	.0119
			IPS (r.)	2.93	.0416
	lateral (r.)	Dorsal attention	IPS (l.)	3.11	.0354
Salience	SMG (r.)	Salience	RPFC (r.)	3.15	.0210
	SMG (l.)	Salience	RPFC (l.)	3.08	.0384
			RPFC (r.)	2.77	.0491
	RPFC (r.)	Visual	Occipital	3.90	.0047
			Medial	3.08	.0384
	RPFC (l.)	Sensorimotor	Lateral (l.)	3.22	.0495
	Anterior insula	Cerebellar	Anterior	3.22	.0495
Dorsal attention	IPS (l.)	Visual	Lateral (l.)	3.77	.0074
			Lateral (r.)	3.11	.0354
Frontoparietal	LPFC (r.)	Frontoparietal	PPC (r.)	3.28	.0410
Language	pSTG (l.)	Default mode	MPFC	3.58	.0146
	pSTG (r.)		MPFC	3.31	.0370
Cerebellum	Anterior	Salience	Anterior insula	3.22	.0495
*(b) Fine motor*					
Default mode	MPFC	Default mode	LP (r.)	3.20	.0491
		Frontoparietal	LPFC (r.)	3.42	.0233
Visual	lateral (l.)	Visual	Lateral (r.)	3.46	.0207
		Dorsal attention	IPS (l.)	3.04	.0279
			IPS (r.)	3.53	.0104
Salience	RPFC (l.)	Salience	SMG (l.)	3.16	.0256
			ACC	3.26	.0234
		Sensorimotor	Lateral (l.)	3.07	.0256
	Anterior insula	Language	IFG (l.)	− 2.99	.0493
		Cerebellar	Posterior	3.36	.0296
	ACC	Cerebellar	Anterior	3.22	.0234
Dorsal attention	IPS (r.)	Visual	Lateral (l.)	3.53	.0160
Frontoparietal	LPFC (r.)	Frontoparietal	PPC (r.)	4.09	.0020
		Default mode	MPFC	3.42	.0117
Cerebellum	Anterior	Salience	ACC	3.22	.0468
	Posterior		Anterior insula (r.)	3.36	.0296
*(c) Visual reception*					
Visual	Lateral (l.)	Visual	Lateral (r.)	3.78	.0064
		Sensorimotor	Superior	− 2.80	.0438
		Dorsal attention	IPS (l.)	3.38	.0107
			IPS (r.)	3.33	.0107
Salience	SMG (l.)	Salience	RPFC (l.)	3.57	.0138
	ACC	Cerebellar	Anterior	3.43	.0230
Dorsal attention	IPS (l.)	Dorsal attention	FEF (l.)	3.18	.0265
		Visual	Lateral (l.)	3.38	.0265
	IPS (r.)	Visual	Lateral (l.)	3.33	.0322
Frontoparietal	LPFC (r.)	Frontoparietal	PPC (r.)	3.77	.0067
		Default mode	MPFC	3.09	.0353
Cerebellar	Anterior	Salience	ACC	3.43	.0230
*(d) Receptive language*					
Default mode	LP (l.)	Salience	SMG (l.)	3.04	.0414
		Language	pSTG (r.)	3.25	.0414
	MPFC	Frontoparietal	LPFC (l.)	3.54	.0079
			LPFC (r.)	3.70	.0079
Visual	Lateral (l.)	Visual	Lateral (r.)	3.73	.0026
		Dorsal attention	IPS (l.)	3.86	.0024
			IPS (r.)	4.03	.0024
Salience	ACC	Language	IFG (l.)	− 3.46	.0208
Dorsal attention	IPS (l.)	Visual	Lateral (l.)	3.86	.0048
	IPS (r.)		Lateral (l.)	4.03	.0025
Frontoparietal	LPFC (l.)	Default mode	MPFC	3.54	.0157
	LPFC (r.)		MPFC	3.70	.0087
Language	IFG (l.)	Salience	Anterior insula (r.)	− 3.46	.0208
	pSTG (r.)	Default mode	LP (r.)	3.25	.0422
*(e) Executive language*					
Default mode	MPFC	Frontoparietal	LPFC (l.)	3.14	.0453
			LPFC (r.)	3.01	.0453
Visual	Lateral (l.)	Visual	Lateral (r.)	3.15	.0195
		Dorsal attention	IPS (l.)	3.74	.0075
			IPS (r.)	3.34	.0158
Dorsal attention	IPS (l.)	Visual	Lateral (l.)	3.74	.0075
	IPS (r.)	Visual	Lateral (l.)	3.34	.0316
		Frontoparietal	LPFC (r.)	3.11	.0338
Frontoparietal	LPFC (r.)	Dorsal attention	IPS (r.)	3.11	.0453
		Default mode	MPFC	3.01	.0453

Significant resting state fMRI network connectivity and relevant motor (a, b), visual (c), and language (d, e) Mullen score interactions with *T* and *p* values on the right. Seed networks and sub regions are displayed in the left two columns, and connected network connectivity regions in the middle. All analyses were corrected for gestational age and biological sex, and *p* values were FDR corrected for multiple comparisons

*MPFC* medial prefrontal cortex, *LP* lateral parietal cortex, *PCC* posterior cingulate cortex, *ACC* anterior cingular cortex, *RPFC* rostral prefrontal cortex, *SMG* supramarginal gyrus, *FEF* frontal eye field, *IPS* intraparietal sulcus, *LPFC* lateral prefrontal cortex, *PPC* posterior parietal cortex, *IFG* inferior frontal gyrus, *pSTG* posterior superior temporal gyrus

Increasing receptive and expressive language ability were independently associated with increased default mode, visual, dorsal attention, and frontoparietal network connectivity, while receptive language was additionally associated with salience and language network connectivity (Fig. 4, Table 3).

Thus, increasing motor and language ability overlapped in default mode, visual, salience, and dorsal attention network connectivity patterns (Figs. 3, 4, Table 3).

While independent *t* tests revealed no significant differences in Mullen raw scores between boys and girls (i.e., gross motor: *F* = 6.984, *p* = 0.701; fine motor: *F* = 1.070, *p* = 0.932; visual reception: *F* = 0.500, *p* = 0.954; receptive language: *F* = 0.011, *p* = 903; expressive language: *F* = 0.698, *p* = 0.988), we did find subtle differences in network development differences with respect to these different skills (Tables S3–S7). Specifically, girls showed more overall connectivity in language domains, with additional language network activation for executive language and visual medial activation in receptive language as well as increased functional connectivity in language and attention networks for visual reception. Boys displayed more overall connectivity in the motor domains with additional activation of visual motor and sensorimotor superior activity for gross motor and cerebellar networks in executive language as well as increased functional connectivity in sensorimotor networks for visual reception. A reference of the distribution of all functional connectivity networks in the infant brain can be found in Figure S1.

## Discussion

Prior neuroimaging studies exploring early development of functional networks have provided a fragmented view, with many studies focusing on either infancy (0–2 years) or later childhood (> 4 years), but few linking these important periods. However, significant gains and refinements in motor control and language abilities occur during ages 2 and 5, laying the foundation for the establishment and refinement of higher cognitive skills and ensuring school readiness. To fill this gap, we applied a data-driven approach in a large neurotypically developing cohort spanning from early infancy to young childhood, demonstrating distinct functional network connectivity patterns with age that overlap with network patterns connected to major visual, motor, and language development.

### Network connectivity with age

Network maturation occurs in specific brain regions associated with goal-directed behavior as well as higher order networks for daily maintenance. While most of these functions are present during early development, their maturation can continue through adolescence (for a review, Barber et al. 2012).

Similarly, we observed functional connectivity patterns for a mixture of networks enabling complex cognitive functions (for example dorsal attention and language networks) as well as higher order networks enabling the increasingly complex daily maintenance (for example default mode and salience networks; Fig. 1, Table 2). This cross-age shift to networks linked to higher-order cognitive processes parallels previous findings about developmental courses of functional connectivity networks.

### Visual function networks

Visual reception describes the ability to interpret information about the surrounding environment that the eyes receive. Visual networks are already present and functioning at birth and are amongst the first to be fully developed reaching an adult-like status (Gao et al. 2017). Similarly, all networks increased in functional connectivity with visual reception in our study.

Increased efficiency and wiring-cost of functional brain networks have been positively associated with increased visual task complexity (Wen et al. 2015). During early development, synaptogenesis precedes later pruning eliminating excess connections (Silbereis et al. 2016), leading to functional network reorganization through more efficient but also more costly network configuration when there is greater demand for cognitive processing. In line with these prior findings, we were able to demonstrate increased functional connectivity with visual networks as well as networks involved in broader higher cognitive functions such as the salience, dorsal attention, and frontoparietal network, supporting optimal visual processing.

### Motor function networks

Work in human and nonhuman primates has indicated that specific brain regions contribute directly to motor function, such as the primary motor cortex, and indirectly, such as supplementary motor areas, to accurate motor execution together with areas involved in cognitive functions (Fink et al. 1997; Rizzolatti and Ruppino 2001; Hanakawa et al. 2003). As a result, a range of networks are required for appropriate motor action including sensorimotor and cerebellar networks, as mirrored in our findings (Fig. 3, Table 3).

Motor function is further typically divided into gross and fine motor abilities. Here, gross motor describes abilities required to control large muscles of the body for functions such as walking, sitting, and crawling, while fine motor function describes the coordination of small muscles, usually involving the synchronization of hands and fingers. In a recent infant study (Marrus et al. 2018), a dynamic subset of resting state motor networks displayed strong relationships with walking and Mullen gross motor scores. These infant/toddler motor associations overlapped with documented adult findings, possibly implying a continuous relationship between functional connectivity and motor skills originating in early development. Specifically, they found gross motor associations with enriched default mode, somatomotor, attention, visual, salience, language, and frontoparietal networks combined for both infant and toddler cohorts—all networks we found showed significant associations with gross motor scores as well (Fig. 3a).

In contrast to the early developing gross motor function, fine motor skills involve a wider range of functions to compose a precisely coordinated movement. As a result, a broader span of functional networks would be required to perform the increasingly complex tasks, as reflected in our results where fine motor scores were associated with more network connectivity than gross motor scores (Fig. 3b).

Interestingly, both gross and fine motor network connectivity patterns overlapped in network connectivity patterns for both primary and non-primary motor areas. This could suggest that while gross and fine motor functions differ, both underlie a consistent network connectivity pattern for motor behavior.

### Language function networks

Support for very early language-related abilities demonstrate that newborns can discriminate between different speech sounds already after birth (for review, Perani et al. 2011). Receptive language describes this ability of understanding words and sentences as well as the meaning of what others say or what is read. Similar to adults (Perani et al. 1996; Binder et al. 2000), speech processing is supported by inferior frontal and temporal brain regions already established at the age of 3 months (Dehaene-Lambertz et al. 2002, 2006). In line with previous findings, our study detected a positive relationship between receptive language scores and a variety of network connectivity patterns (Fig. 4a).

In contrast to the more passive ability receptive language requires, expressive language describes the ability to actively put thoughts into words and sentences, in a senseful and grammatically accurate way. When learning how to speak, one of the predominant developmental transition is the lateralization of the brain. Studies in adults have shown that some systems, like the primary sensory systems require fine-tuned integration between the hemispheres while other higher-order functions like language typically show asymmetry (Binder et al. 2000; Toga and Thomspson 2003; Friederici and Alter 2004; Stark et al. 2008; Perani et al. 2011).

These language-relevant brain areas are structurally and functionally connected (Dubois 2006), with a set of lateral brain regions in the left frontal, temporal, and parietal cortices being activated in previous studies during linguistic processing (Perani et al. 2011). This is paralleled by our findings, as frontal, temporal, and parietal regions increased with expressive language scores and all network connectivity patterns included bilateral and/or left hemisphere regions only (Fig. 4b).

While language networks are functional from infancy on, their functional network connectivity starts bilateral and then develops towards unilaterally with development. Interestingly, we are able to support this notion. Similarly to motor functions, both receptive and expressive language network connectivity patterns overlapped in network connectivity patterns, namely the default mode, visual, salience, dorsal attention, and frontoparietal network connectivity (Table 3, Fig. 4). In addition, as language function becomes more complex (from receptive to executive), these overlapping networks become more left hemisphere dominant.

### Possible motor and language contributions to other network development

The more complex the ability was, the more functional connectivity networks were recruited (i.e. gross to fine motor and receptive to expressive language). In addition, both motor and language network connectivity patterns overlapped in network connectivity patterns for the default mode, visual, salience, and dorsal attention networks. These overlaps in network connectivity could implicate their overarching contribution to each other’s and higher cognitive development, as for example gross motor skill development in infants and toddlers is predictive of future cognitive outcomes (Marrus et al. 2018). The many positive and negative brain–behavior relationships we observed throughout our study could further imply that increases and decreases in network-level connectivity may underlie the characteristic complexity needed for both motor and language functioning.

## Conclusion

Our study investigated the typical development of functional network connectivity during infancy and childhood, as well as their relationship with the emergence and establishment of cognitive abilities. With age, these behaviors become more and more refined and occur with increasing complexity, starting with gross to fine motor skills and finally progressing to more complex cognitive abilities. Our findings capture the developmental timeline of these early visual, motor, and language skills, as well as their gradual development towards adult-like networks. We further found a distinct brain–behavior relationship involving the default mode, visual, salience, and dorsal attention network, supporting both motor and language refinement. This could implicate a contribution to the maturation of cognitive functions during infancy and childhood development.

While our study focused on neuro-typically developing children, our findings will be important for the understanding and detection of abnormal development. Children may fail to reach important early motor and language milestones for a variety of reasons, including neurodevelopmental disorders (e.g., autism spectrum disorder), prenatal insults (e.g., drug exposure), or other early environmental adversity (e.g., severe neglect). Our results provide a normative template to which these children may be compared to and enabling early deviations to be identified. Future work is needed to understand how these developmental processes are linked to the emergence of cognitive functions and how they are guided by complex underlying biological mechanisms.

## Electronic supplementary material

Below is the link to the electronic supplementary material.

Figure S1 Overview over resting state fMRI connectivity networks. From left to right: cerebellar (anterior, posterior), default mode (MPFC, medial prefrontal cortex; left and right LP, lateral parietal cortex; PCC, posterior cingulate cortex), dorsal attention (left and right FEF, frontal eye field; left and right IPS, inferior frontal gyrus), fronto-parietal (left and right LPFC, lateral prefrontal cortex; left and right PPC, posterior parietal cortex), salience (left and right anterior insula, left and right RPFC, rostral prefrontal cortex; left and right SMG, supramarginal gyrus), sensorimotor (superior, left and right lateral), visual (medial, occipital, left and right lateral) and language (left and right IFG, inferior frontal gyrus; left and right pSTG, posterior superior temporal gyrus) networks (TIFF 279 kb)
