# Supplementary figures and images for: Functional connectivity correlates of infant and early childhood cognitive development
Source: Brain Struct Funct. 2020 Feb 15;225(2):669–81. doi: 10.1007/s00429-020-02027-4 (PMC7046571; doi:10.1007/s00429-020-02027-4)

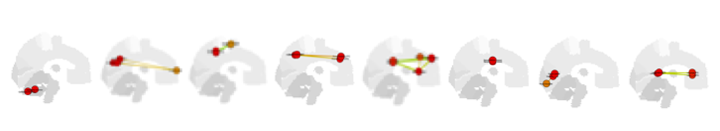

Supplement: Supplementary file 1 — Figure S1 Overview over resting state fMRI connectivity networks. From left to right: cerebellar (anterior, posterior), default mode (MPFC, medial prefrontal cortex; left and right LP, lateral parietal cortex; PCC, posterior cingulate cortex), dorsal attention (left and right FEF, frontal eye field; left and right IPS, inferior frontal gyrus), fronto-parietal (left and right LPFC, lateral prefrontal cortex; left and right PPC, posterior parietal cortex), salience (left and right anterior insula, left and right RPFC, rostral prefrontal cortex; left and right SMG, supramarginal gyrus), sensorimotor (superior, left and right lateral), visual (medial, occipital, left and right lateral) and language (left and right IFG, inferior frontal gyrus; left and right pSTG, posterior superior temporal gyrus) networks (TIFF 279 kb) [file 429_2020_2027_MOESM1_ESM.tiff]
